# Large Animal Models of Heart Failure: Reduced vs. Preserved Ejection Fraction

**DOI:** 10.3390/ani10101906

**Published:** 2020-10-18

**Authors:** Christopher J. Charles, Miriam T. Rademaker, Nicola J. A. Scott, A. Mark Richards

**Affiliations:** 1 Christchurch Heart Institute, Department of Medicine, University of Otago, Christchurch, Christchurch 8011, New Zealand; miriam.rademaker@otago.ac.nz (M.T.R.); nicola.scott@otago.ac.nz (N.J.A.S.); mark.richards@cdhb.health.nz (A.M.R.); 2 Cardiovascular Research Institute, National University Heart Centre Singapore, Singapore 119074, Singapore; 3 Department of Surgery, Yong Loo Lin School of Medicine, National University of Singapore, Singapore 119077, Singapore

**Keywords:** heart failure, animal model, porcine, ovine, HFrEF, HFpEF, ejection fraction

## Abstract

**Simple Summary:**

Human heart failure (HF) is a complex clinical syndrome that can be caused by a variety of diseases. While long-term high blood pressure and heart attacks are major contributing factors, there can be many diseases of the heart and circulation that contribute to the development of HF. Although there have been salutary improvements in the medical management of HF over the last 30 years, ongoing ill effects of living with the syndrome and the persistently high death rates mean there is an irrefutable need for new and improved treatment options. Well-characterized animal models have contributed, and continue to contribute, much to the advancement of clinical care. This review will summarize the main large animal models of HF developed to date. Studies utilizing these large animal models are an essential step leading to the development of novel pharmaceutical and device-based therapies before they can undergo definitive clinical trials. This review will discuss the various benefits of different large animal models of HF and highlight some key deficiencies to date. There is clearly a need for ongoing development of clinically relevant large animal models of HF.

**Abstract:**

Heart failure (HF) is the final common end point of multiple metabolic and cardiovascular diseases and imposes a significant health care burden worldwide. Despite significant improvements in clinical management and outcomes, morbidity and mortality remain high and there remains an indisputable need for improved treatment options. The pathophysiology of HF is complex and covers a spectrum of clinical presentations from HF with reduced ejection fraction (HFrEF) (≤40% EF) through to HF with preserved EF (HFpEF), with HFpEF patients demonstrating a reduced ability of the heart to relax despite an EF maintained above 50%. Prior to the last decade, the majority of clinical trials and animal models addressed HFrEF. Despite growing efforts recently to understand underlying mechanisms of HFpEF and find effective therapies for its treatment, clinical trials in patients with HFpEF have failed to demonstrate improvements in mortality. A significant obstacle to therapeutic innovation in HFpEF is the absence of preclinical models including large animal models which, unlike rodents, permit detailed instrumentation and extensive imaging and sampling protocols. Although several large animal models of HFpEF have been reported, none fulfil all the features present in human disease and few demonstrate progression to frank decompensated HF. This review summarizes well-established models of HFrEF in pigs, dogs and sheep and discusses attempts to date to model HFpEF in these species.

## 1. Introduction

Heart failure (HF) is the final common pathway of numerous metabolic and cardiovascular diseases. The underlying etiology of HF is diverse, with many different pathological processes initiating and progressing the downward spiral of cardiac function in developing HF. The common fundamental defect is a decreased ability of the heart to provide sufficient output to support the normal functions of the tissues because of impaired filling and/or ejection of blood. In particular, when overt HF has developed, a number of hemodynamic, neurohumoral, renal and volume-retaining features are manifested which treatment may alleviate but not cure the underlying cardiac impairment. As the primary symptoms of HF include dyspnea, fatigue, exercise intolerance and retention of fluid in the lungs and peripheral tissues [1], it is important that any animal model purporting to replicate human HF displays at least some of these features.

HF is a debilitating and costly syndrome projected to eventually afflict 1 in 5 individuals currently 40 years of age [2], with prevalence increasing due to improved long-term survival in coronary heart disease and hypertension within ageing populations, and burgeoning contributions from risk factors such as diabetes and obesity [3]. Although there have been major improvements in clinical management and outcomes, morbidity and mortality remain high worldwide, with the latter approximately 10% at 1 month, 20–30% at 12 months and up to 60% at 5 years after initial diagnosis [4]. Thus, there is an irrefutable need for new and improved treatment options. Whilst many patients have HF with reduced ejection fraction (HFrEF), namely an EF ≤40%, a significant proportion of HF patients have preserved EF (HFpEF). HFpEF patients’ hearts are characterized by a reduced ability to relax but EF remains above 50%, hence “preserved” [5]. Early reports from epidemiological reports published in the mid-2000s suggested that prevalence and mortality from HFpEF was similar to those for HFrEF [6,7]. Over the ensuing decade, there were progressive reports suggesting that both prevalence and mortality were not so equivalent as highlighted by the MAGGIC meta-analysis [8]. A more recent prospective multiethnic study from our laboratories shows that HFpEF accounts for ~30% of all HF cases in parallel cohorts recruited in both Singapore and New Zealand. Compared with HFrEF, HFpEF has greater prevalence in females and is characterized by older age and a predominant background of hypertension (78%) [9]. While mortality measured at two years was lower than in HFrEF, it is still high at 14%, with a composite outcome of all-cause mortality or HF hospitalization occurring in 35% of HFpEF patients.

To date, clinical trials in HFpEF patients have failed to show any reduction in mortality. A 2018 meta-analysis of 25 trials encompassing data from >18,000 patients with HFpEF showed no beneficial effects on mortality or hospitalization for HF of any of the key pharmaceutical agents that are effective in HFrEF, namely angiotensin-converting enzyme inhibitors, angiotensin receptor blockers, beta blockers or mineralocorticoid antagonists [10]. The absence of evidence-based effective treatments for HFpEF highlights a critical unmet need. One of the key impediments to developing novel and effective therapeutic agents for the treatment of HFpEF is the dearth of large animal preclinical models of HFpEF. Large animal models are an essential step in bench-to-bedside translation, as they allow detailed instrumentation and extensive imaging and sampling protocols [11]. Although several large animal models of HFpEF have been reported recently [12], few, if any, fulfil all the features present in human disease and few demonstrate progression to frank decompensated HF.

This review will summarize important large animal models of HF which have underpinned multiple preclinical studies leading to the development and translation of key medical and device-based therapies for HFrEF. This paper will then review attempts to develop large animal models of HFpEF and highlight some of the deficiencies of models to date, highlighting the ongoing need for more research into developing representative, clinically relevant models of HFpEF.

## 2. Models of HFrEF

In addition to the two main models of HFrEF discussed below, namely models of myocardial ischemia/infarction and the pacing model of HF, a number of other less common large animal models have been described in the literature. These include volume overload, such as surgically induced mitral regurgitation [13] or creation of an arteriovenous fistula [14], and cytotoxic models such as administration of doxorubicin to pigs and sheep [15,16]. As these are relatively infrequently used models, they will not be discussed further here but have been included in reviews by several authors recently [1,17].

### 2.1. Models of Acute Myocardial Ischemia/Infarction

Historically, dogs have been used in many early studies as the large animal species for modeling heart disease, especially for the study of myocardial ischemia and arrhythmia following coronary occlusion as exemplified by the work of Reimer et al. [18,19]. However, a key shortcoming of the canine model is the presence of extensive epicardial coronary collateral circulation in the epicardium, which results in great variability of infarct size between individual animals. In addition to this key anatomical difference between the human and canine coronary circulation, local ethical and cultural reasons have resulted in a decrease in the use of dogs as experimental models. In more recent decades, pigs have gained popularity for use in models of cardiovascular disease due to the comparability of porcine and human physiology, coronary anatomy (with relatively underdeveloped coronary collaterals) and heart/body size. Likewise, sheep have many of the same attributes as pigs and so are also regarded as excellent subjects for modeling of human cardiovascular disease [17]. There is extensive literature on large animal models of acute myocardial infarction (MI). This review will cite only a few studies that illustrate specific points of interest.

**Figure 1:**
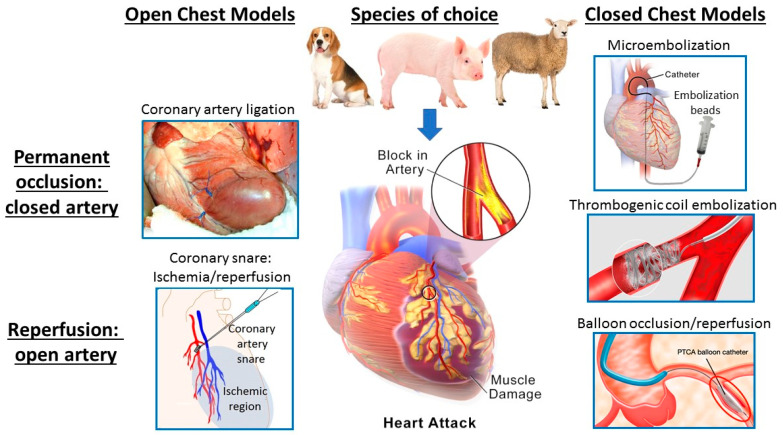
Variations of large animal models of acute myocardial ischemia/infarction showing species of choice and key variations in methodology ranging from open chest to closed chest and permanent occlusion to reperfused, open artery.

Myocardial infarction can be induced in large animals by a myriad of methods spanning open chest to closed chest and closed artery to open artery, as shown in Figure 1.

An example of a closed-chest MI model is the intracoronary injection of agents such as microsphere beads down selected arteries under fluoroscopy, causing microembolization of coronary arterioles and resulting in patchy diffuse ischemia with necrosis of tissue subtended by the small vessels in which the microspheres become lodged [20]. Other closed-chest models include angiographic catheterization of specific coronary arteries such as the left anterior descending (LAD), again under fluoroscopy, followed by deployment of thrombogenic coils which cause clotting within 1–2 min resulting in permanent occlusion of the coronary artery with a downstream infarct in the region of myocardium previously perfused by that artery [21]. This method in sheep was compared to closed-chest balloon occlusion for 90 min followed by balloon deflation and removal, resulting in complete reperfusion of the coronary artery—hence, an open-artery or ischemia-reperfusion model in the closed chest [21]. These variations on a theme contrast with the more traditional open-chest models whereby the animals undergo an open-chest surgical procedure such as sternotomy or lateral thoracotomy with occlusion by ligation of the selected coronary artery followed by closure of the chest and post-surgical recovery. These models typically induce permanent occlusion (closed artery) achieved by ligating or tying a permanent suture around the coronary artery [22]. Alternatively, the artery can be temporarily snared (usually for ~90 min if infarction is intended) with the snare released in order to reperfuse the myocardium, followed by surgical closure of the chest. Of note, ischemia reperfusion during an open-chest procedure significantly extends the anesthetic time required. As an alternative, we have also investigated a model whereby a coronary artery snare was placed at time of surgery with the ends exteriorized and then the snare tightened four to six days later in the conscious (but sedated and analgesed) sheep, thereby allowing measurement of cardiac sympathetic nerve activity (CSNA) during acute ischemia/infarction [23,24]. Actuating the snare in the conscious animal is essential for these studies as it is not possible to record CSNA in the fully anesthetized animal as all classes of general anesthetic agents suppress cardiac sympathetic nerve firing. Thus, there is a variety of ways of inducing MI in large animal models, with the ability to vary the model depending on the specific clinical scenario to be modeled and questions to be answered. As all of the above sheep models were performed in our Christchurch Heart Institute (CHI) laboratory and all were targeting a similar territory of the LAD (approximately at the level between the first and second diagonal branches), we can compare the resultant infarct sizes and degree of left ventricular (LV) dysfunction. In summary, the rank order of LV dysfunction is as follows: microembolization < balloon occlusion/reperfusion < permanent occlusion by either thrombogenic coil or ligation [20,21,22]. Microembolization does not result in a clearly defined infarct (instead diffuse patchy ischemia), while infarcts from permanent occlusion (closed-artery models) result in larger infarcts than equivalent targeted ischemia/reperfusion (open-artery) models [21,22].

Of course, none of these models reflects the predominant underlying pathology of the majority of acute MIs in patients, namely, progressive atherosclerotic narrowing of coronary arteries complicated by sporadic rupture of vulnerable atherosclerotic plaques triggering thrombotic occlusion of the artery. However, each of these models has utility and each has advantages and disadvantages. While microembolization is of relatively low complexity—only requiring cardiac catheterization procedures, it is comparatively less reproducible, less precise, non-reversible and leads to a relatively small degree of LV dysfunction. However, some investigators have employed multiple episodes of embolization, usually several weeks apart [25]. Whilst this does result in further reductions in LVEF, it also necessitates multiple interventions, thus negating the simplicity of the model. Balloon occlusion/reperfusion has the two very clear advantages of being a closed-chest model, therefore mitigating the significant impact of major surgical intervention, and allowing reperfusion of the artery—thus modeling the large majority of present day patients who undergo either spontaneous reperfusion or clinical revascularization by percutaneous coronary intervention within several hours of MI. However, placement of the balloon under fluoroscopic guidance tends to be less precise than open-chest visualization of the coronary artery anatomy and hence the area at risk may be more variable. Open-chest ligation allows precise selection and placement of the site of occlusion but does of course require concurrent major surgical intervention to open the chest and, unless a snare type arrangement is deployed, ligation results in a permanently non-perfused artery. Nevertheless, this does have the advantage of maximizing infarct size and thus is often preferred as a first pass model for assessing the efficacy of novel therapeutic agents in reducing infarct size.

Taken together, it is important to realize that although all of these models can and do result in clear infarcts and some degree of LV dysfunction, the dysfunction is often mild to moderate due to the need to balance the degree of ischemic damage and resultant infarct size against the propensity for animals to suffer lethal arrhythmia [21,22,24]. Although all models result in acute changes in hemodynamics and neurohumoral activation over the first 24 h consistent with acute MI, the longer-term (1–4 weeks) effects to raise key hemodynamic indices of HF such as left atrial pressure (LAP) and circulating levels of HF biomarkers such as B-type natriuretic peptide (BNP) are modest at best [22]. Therefore, the majority of large animal models of coronary ischemia should be regarded more as models of acute MI with and without mild-to-moderate LV dysfunction, rather than HF per se.

### 2.2. Pacing Models of HF

A widespread method for inducing HF of varying degrees is rapid cardiac pacing. Dogs [26], pigs [27] and sheep [28] have all been used in rapid pacing models of HF. As with models of MI, there are many variations to the methodology employed—with pacing wires placed in either the atria or ventricles (or sometimes multi chamber pacing) and pacing rates (typically 180–240 bpm depending on the species) continued for a range of time generally from 1 to 5 weeks. Rapid pacing usually leads to biventricular dilatation and is thought to induce an intrinsic loss of myocardial contractility, further supported by the observation that contractile reserve in response to inotropic drugs is severely reduced [29]. However, unlike typical HFrEF, the reduction in LV function induced in rapid cardiac pacing models is not generally accompanied by changes in LV mass and fibrosis [27,30].

**Figure 2:**
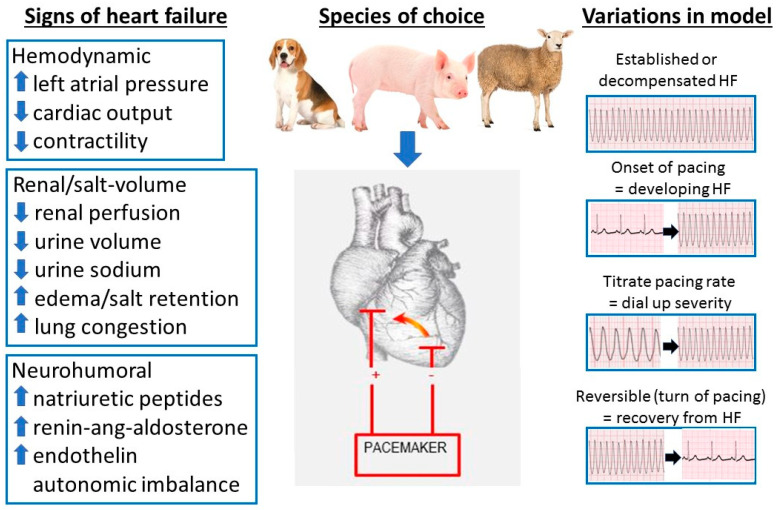
Variations of the large animal model of pacing-induced heart failure showing species of choice and key integrative hemodynamic, renal and neurohumoral signs of heart failure.

We have utilized the ovine pacing model of HF in our CHI laboratory for over three decades and so have extensive experience in variations of the pacing model used to answer different scientific questions modelling various clinical scenarios, as shown in Figure 2.

Our main iteration of the pacing model involves thoracotomy to stitch pacing wires subepicardially to the free wall of the LV, which at the same time allows extensive instrumentation of various chambers of the heart and major vessels with a combination of fluid filled and hi-fidelity pressure tipped transducers. After two weeks surgical recovery, we commence pacing at ~225 bpm for a period of 7 days (although this duration can be varied depending on the subsequent experimental protocols). Measurements made in the conscious animal reveal that this model of pacing-induced HF closely replicates the key peripheral manifestations of severe low cardiac output human HF. This includes hemodynamic changes consistent with moderate to severe decompensated HF including rises in LAP—a key indicator of HF severity/decompensation—to levels in the range of 20–30 mmHg, and falls in cardiac output and contractility [28]. Renal perfusion pressure is appreciably reduced, contributing to significant salt and water retention. There is also significant activation of multiple neurohumoral axes including the natriuretic peptides (with levels of BNP and atrial natriuretic peptide (ANP) consistent with severe decompensated HF), renin–angiotensin–aldosterone system and endothelin [28,31]. With this model, the induction of HF has negligible attrition rates, and produces a stable and extremely reproducible state. In addition, the large body size and blood volume of the sheep permits comprehensive instrumentation to provide diverse and multiple hemodynamic measurements and allows repeated blood and urine sampling—all of which are vital in capturing an integrated picture of the biological effects of an agent. Hence, we have used this model in numerous translational studies investigating the pathophysiology of HF and in the development and refinement of novel HF therapies [28,31,32,33,34].

A number of key variants on the pacing model make it particularly useful for studying different clinical settings (Figure 2). Unlike most models, the severity of HF can be titrated by varying the pacing rate, thereby allowing investigation in varying degrees of cardiac dysfunction either in separate protocols or even in the same animal [31]. For example, pacing at 180 bpm for 4–7 days results in mild HF, and then turning the pacing rate up to 225 bpm “decompensates” the HF to more severe levels, giving an ability to, in effect, “dial up” the degree of HF by adjusting the pacing rate up (or down) with hemodynamic, neurohumoral and renal indices shifting in parallel [31,32]. We have also used the model to assess the effects of treatments on slowing the progression of HF by administering agents from the onset of pacing rather than waiting until severe HF is already established, as demonstrated by the effects of the peptide hormone urocortin 1 to repress progression to overt heart failure [33]. A unique attribute of the pacing model is that it is essentially reversible, as termination of pacing results in rapid (within 1–2 days) recovery of virtually all hemodynamic, renal and neurohumoral indices to normal [28,31]. Not only does this allow study of physiological responses to recovery from acute decompensated HF, but also allows multiple episodes of HF in the same animal and hence reduce “noise” in the data from inter-animal variability whilst allowing observance of the ethical goals of “reduction” in total numbers of animals required for the study. Lastly, in instances where less monitoring of hemodynamic indices is necessary, instrumentation can be greatly simplified by a less surgically invasive approach of fluoroscopic placement of a right ventricular pacing lead via a peripheral vein such as the jugular. Pacing the right ventricle at similar rates and duration (for example 225 bpm for 7 days) also induces a similar degree of HF as that seen with LV pacing with less surgical demand on the sheep, hence ethical “refinement” of procedures [34]. Taken together, the ability to “dial up” severity of hemodynamic, renal and neurohumoral indices and study the onset and recovery from HF in a highly predictable and time-dependable fashion makes this an excellent model, particularly of decompensated HF.

One limitation of this pacing model of HF is that the pacemaker is required to stay on throughout the studies, as cessation of pacing results in relatively quick return (within 1–2 days) of the majority of indices towards pre-pacing values. This can confound some measurements of hemodynamic indices. It also renders the model unsuitable for testing of some devices such as pacemakers and those that alter heart rate directly. Some authors have reported another variation of the pacing model of HF which does not require keeping the pacing on during study. An example is Ramchandra et al. who subjected sheep to long-term (8–12 weeks) right ventricular pacing at 200–220 bpm [35]. Each week, the pacemaker was temporarily turned off to allow echocardiographic assessment of LVEF. Once LVEF was reduced <40%, the sheep were deemed to be in HFrEF and other studies commenced with the pacemaker remaining off. LVEF in the HF sheep was 36 ± 2% compared with 78 ± 2% in normal and the fractional shortening was decreased to 18 ± 1% compared with 35 ± 1% in normal. However, it is not clear whether other key indices indicative of HF such as filling pressures and BNP are raised in this model nor the stability of the dysfunction for a prolonged period of time after cessation of pacing [35].

## 3. Models of HFpEF

As stated in the introduction, one of the key impediments to developing novel and effective therapeutic agents for the treatment of HFpEF is the scarcity of large animal preclinical models of this condition. Although several large animal models of HFpEF have been reported recently [12], few, if any, fulfil all the features present in human disease and few demonstrate progression to frank HF. The unmet need for models of HFpEF allowing both elucidation of the underlying biology of this syndrome and providing a testing platform for candidate novel therapies has triggered interest in developing large animal models of HFpEF. Efforts in this direction to date have had varying degrees of success.

The widely used rodent deoxy-corticosterone acetate (DOCA)–salt rodent model has been up-scaled to pigs, with variable results [36,37]. Pigs treated with DOCA show increased mean arterial pressure (approximately 20 mmHg compared to control pigs) LV mass and wall thickness and greater left atrial volumes compared with control pigs [36]. Under dobutamine stress, LVEF and LV end systolic volume (LVESV) demonstrated normal contractile reserve but there are some differences in LV end diastolic volume (LVEDV) and cardiac index indicative of early-stage HFpEF, but no change in fibrosis as assessed by T1 mapping by magnetic resonance imaging (MRI) [36]. If the DOCA-induced hypertension is combined with high salt, sugar, fat and cholesterol diet, with tail cuff pressures measured at 40 mmHg higher than controls, then pigs develop concentric LV hypertrophy (LVH) and dilation of the left atrium with no fall in LVEF or HF features at rest [37]. There is some evidence of perturbation of the pressure–volume relationships, but LV end diastolic pressure (LVEDP) was not elevated unless pigs were further challenged with pacing and dobutamine. A recent study invoked an even more complex approach by modelling multiple comorbidities including hypertension and kidney dysfunction (induced by renal artery embolization), diabetes mellitus (induced by streptozocin injection) and hypercholesterolemia (produced by a high fat diet) in association with a high salt/sugar diet in pigs [38]. After a period of six months, pigs showed coronary endothelial dysfunction, systemic inflammation, and a rise in reactive oxygen species with disruption of nitric oxide. In addition, they displayed cardiomyocyte stiffness and cardiac fibrosis. The authors claimed that their model was a clinically relevant model of LV diastolic dysfunction and a precursor for HFpEF, but there was no evidence presented that any pigs progressed to overt HF. Nonetheless, this model may be a good model to study stable HFpEF with multiple co-morbidities that does not decompensate to overt HF.

Thus, whilst a number of large animal models have been proposed as models of HFpEF, none fulfil all the features present in human disease. However, human HFpEF is a complex syndrome with multiple contributing etiologies which occur in varying degrees alongside differing severity of comorbid conditions. Animal models invariably model some but not all aspects of pathology, and therefore researchers need to select combinations of pathological features according to the specific aims of their study. Many of the models noted above replicating preclinical disease with no objective evidence of HF thus, they more accurately represent early stages of the disease process and not HFpEF per se.

### Pressure Overload Models of HFpEF

A large number of investigators have employed variations of pressure overload inducing LVH often with the goal of developing/characterizing large animal models of HF. Various iterations of aortic constriction, usually by surgically inducing an acute constriction on the aorta in close proximity to the heart, have long been used as rodent models of pressure overload LVH and HF. For decades, investigators have tried to upscale these techniques to large animal models. In the mid-1990s, our CHI laboratory attempted such a model in sheep [39]. We induced acute aortic constriction at a level just proximal to the branching of the renal arteries resulting in severe pre-morbid (euthanized) or terminal HF in all sheep with a rapid but variable (median 15 days) deterioration to HF/death [39]. The sheep all displayed significant LVH and plasma BNP levels rose to levels indicative of moderate-severe HF in the days before the ethically-determined (pre-morbid) euthanasia time-point or death. Despite the clear cut development of symptomatic, BNP-confirmed HF, the unpredictable timing of the onset and deterioration of HF and in ability to achieve a stable time window for interventional tests meant we did not progress with the model for the study of novel therapeutics.

Other examples of pressure overload models in large animals include various attempts at aortic constriction in pigs. A study inducing acute onset aortic constriction at a level distal to the carotid bifurcation resulted in rapid development of LVH within 7 days in association with a reduction in LVEF and what the authors described as acute HF, with attrition of approximately 20% of the animals [40]. The surviving pigs were then monitored for a further 7 weeks with their hearts undergoing remodeling to a compensated phase of concentric LVH with cardiac function restored [40]. Other authors banded the ascending aorta of minipigs (25–30 kg), but showed no changes in LV mass, left atrial volume or LVEDP over 20 weeks and did not report plasma BNP, thereby failing to describe any objective evidence of HF [41]. The Mayo group showed that renal wrapping in old dogs induced chronic hypertension with secondary LVH and fibrosis with impaired LV relaxation, but no increase in LV diastolic stiffness which they claim mimics HFpEF [42]. However, the requirement for old animals (8–13 years old) makes this model relatively impractical and expensive.

Rather than inducing acute aortic constriction, it was hypothesized that a gradual and progressive constriction of the aorta may better model of human hypertensive heart disease that may preserve systolic function and improve survival. Thus, the Spinale group at the University of South Carolina developed a porcine model of progressive LV pressure overload (LVPO) [43]. In this model, an inflatable cuff is placed around the aortic root of young growing pigs (weighing 15 kg at time of implantation) and is inflated in a stepwise fashion to give pressure gradients across the constriction of approximately 20, 40, 60 and 80 mmHg over consecutive weeks. Over the four-week course of progressive LVPO, the pigs develop significant LVH (two-fold increase) with no change in LVEDV or LVEF [43]. In contrast, LV diastolic function is significantly impaired, as evidenced by elevated LVEDP and reduced active relaxation rates (tau, negative dP/dt) and a three-fold increase in regional myocardial stiffness. Thus, this porcine model of LVPO results in significant LVH with impaired diastolic function but with no significant compromise of LV systolic function. In addition, total myocardial collagen content increased by approximately 50% in association with a greater insoluble collagen fraction—indicative of reduced solubility [43]. Although they did not report symptomatic HF, this model appears to be an excellent substrate for HFpEF with underlying etiology of hypertensive heart disease.

Our Cardiovascular Research Institute laboratory in Singapore recently set about establishing a model of HFpEF by enhancing the porcine model of progressive LVPO [43]. We utilized the same progressive increments in aortic constriction, titrating the volume injected into the inflatable cuff to achieve pressure gradients of approximately 20, 40, 60 and 80 mmHg over the first four weeks. We characterized HF in this model with advanced cardiometabolic imaging using cardiac magnetic resonance imaging (CMRI) along with hyperpolarized carbon-13 magnetic resonance (HP^13^CMR) spectroscopy [44]. This porcine HFpEF model demonstrates significant LVH (doubling of LV mass) without LV dilation, similar to that previously reported [43]. Unique features of our model that document HF include comprehensive serial imaging (echocardiography and MRI) clearly demonstrating global ventricular fibrosis by MRI T1 MOLLI, significant increase in plasma BNP, and significantly elevated pulmonary capillary wedge pressure and LVEDP [44]. Features indicative of overt HF were apparent after 4–6 weeks of the study, not in the early stages of aortic constriction. Hyperpolarized ^13^C-MR studies also showed increased alanine transaminase and pyruvate dehydrogenase activity in these HFpEF pigs. Pigs exhibited incipient HF with frequent progression to overt decompensated HFpEF manifested in clinical symptoms, raised LV filling pressures and, in some pigs, frank pulmonary edema [44]. Thus, we have described a steadily progressive, but sufficiently stable and controllable (via titration of cuff/constriction) model of LVPO with novel data evidencing fibrosis based on MRI T1 MOLLI mapping, elevated filling pressures, metabolic changes and progression to frank heart failure with prolongation of the model. This is a clinically relevant model of HFpEF with clear utility in advancing knowledge of underlying pathophysiology of HFpEF and testing novel candidate therapies.

Not all laboratories have access to imaging such as MRI for large animals. In 2018, the Working Group on Myocardial Function of the European Society of Cardiology published a position paper entitled “An integrative translational approach to study heart failure with preserved ejection fraction” [45]. This comprehensive position paper discusses most appropriate approaches to developing and characterizing large animal models of HFpEF. With respect to hemodynamics, the gold standard is to assess LV compliance and relaxation in vivo but this requires anesthesia, surgical preparation, mechanical ventilation and the open chest deviates from physiology. For non-invasive imaging assessment, echocardiography is often the first choice. Most clinical assessments performed by echocardiography can readily be utilized in large animals including assessment of flows, dimensions and strain. Again, not all animals are amenable to echocardiography examination in the conscious state and often require at least light sedation. Traditional measurements such as E/E’ ratio can be useful in assessment of diastolic dysfunction but caution is necessary in trying to interpret such indices in isolation [45].

## 4. Conclusions

Human HF is a complex clinical syndrome resulting from a variety of disease processes. Chronic hypertension and ischemic heart disease are common etiologies but many other causes may contribute to the development of HF either alone or, more commonly, in combination. Animal models invariably model some but not all aspects of pathology. Thus, researchers need to select combinations of pathological features according to the specific aims of their study. Ultimately, the choice of the most appropriate model is determined by the specific research questions to be addressed. With respect to species of choice, of the main species utilized around the world, namely canine, porcine and ovine, they are often equally valid for most HF-related research questions and the main determinants in choosing any particular species are often local including practical concerns such as accessibility of species, investigator experience, available facilities, funding and local regulations. Many of the models noted above, especially the ischemic/MI HFrEF and many of the putative HFpEF models, only replicate preclinical disease with no objective evidence of HF, and therefore they more accurately represent early stages of the disease process and not HF per se. Well-characterized animal models have much to offer to the advancement of clinical care. Indeed, investigation of molecular pathways in both early and late stages of HF can identify novel therapeutic targets for intervention or assessments of biomarkers. Further, studies in large animal models usually provide important preclinical proof of concept for novel therapies and underpin progression to clinical trials. There is a clear need for ongoing development of clinically relevant large animal models of HF, particularly models of HFpEF.
